# An update on current understanding of the epidemiology and management of the re-emerging endemic Lassa fever outbreaks

**DOI:** 10.1097/JS9.0000000000000178

**Published:** 2023-03-24

**Authors:** Sumira Malik, Jutishna Bora, Archna Dhasmana, Shristi Kishore, Sagnik Nag, Subham Preetam, Priyanka Uniyal, Petr Slama, Nobendu Mukherjee, Shafiul Haque, Sarya Swed

**Affiliations:** a Amity Institute of Biotechnology, Amity University Jharkhand, Ranchi, Jharkhand; b Himalayan School of Biosciences, Swami Rama Himalayan University; c Department of Pharmaceutical Sciences and Technology, Sardar Bhagwan Singh University, Dehradun, Uttarakhand; d Department of Biotechnology, School of Biosciences & Technology, Vellore Institute of Technology (VIT), Vellore, Tamil Nadu; e Department of Microbiology, West Bengal State University, Kolkata, West Bengal, India; f Laboratory of Animal Immunology and Biotechnology, Department of Animal Morphology, Physiology and Genetics, Faculty of AgriSciences, Mendel University in Brno, Brno, Czech Republic; g Institute of Advanced Materials, IAAM, Ulrika, Sweden; h Department of Health Sciences, Novel Global Community Educational Foundation, Hebersham, New South Wales, Australia; i Research and Scientific Studies Unit, College of Nursing and Allied Health Sciences, Jazan University, Jazan, Saudi Arabia; j Centre of Medical and Bio-Allied Health Sciences Research, Ajman University, Ajman, United Arab Emirates; k Faculty of Medicine, Aleppo University, Aleppo, Syria

*Dear Editor*,

Lassa virus (LASV) is a zoonotic, acute, fatal, disease-causing virus, a causative agent of Lassa fever (LF) which is a type of viral hemorrhagic fever that is endemic in various parts of West Africa including Liberia, Nigeria, Guinea, and Sierra Leone. Past research indicated that although the first primary incidence of LF was noticed in 1969, the zoonotic transfer of this viral agent to humans has been occurring for more than a centenary. LASV or Lassa virus is a single-stranded, encapsulated, bipartite RNA virus that predominantly corresponds to the family of *Arenaviridae*. This virus resembles a circular or round morphology with a usual approximate diameter of 110–130 nm. LASV requires an intricate nucleotide diversity among strains, which is intertwined with the subgroups of strains across geographical locations. As a result, six main LASV clades or lineages have been identified: clades I–III in Nigeria; clade IV covers up the countries of Sierra Leone, Guinea, and Liberia; and clade V in southern Mali; and a more contemporary clade VI, emerging from Togo. The capacity of these strains to change over time is a special feature^1^. The ‘multimammate rat’ *Mastomys natalensis* is suspected to be the animal reservoir for LASV. It’s a rodent from the *Mastomys* genus, which is common and reproduces a majority in West Africa^2^. Primary or first infection in humans happens because of direct or indirect interaction with rodents that have LF. Exposure to the urine, feces, blood, or flesh of *Mastomys* rats infected with LASV is the cause of LASV infection in humans^3^,^4^.

A considerable section of LF cases is primarily instituted by rodent-to-human LASV transmissions associated with close proximity with infected animals or animal excreta, needless to say, there are high possibilities of human-to-human transmissions. Even though LASV infection can cause symptoms in patients, it is thought to cause asymptomatic infection in its natural animal reservoirs. The peak of the LF in West Africa is during dry seasons and the lowest is in the wet season. The incubation period for LF is somewhere between 7 and 21 days after exposure. The WHO estimates that 80% of LASV-infected people have no symptoms, whereas 20% of infected people have severe, multisystem illnesses.^[44]^ Since the early symptoms and indications of LF are frequently mild and similar to those of other enteric fevers, LF clinical diagnosis can be difficult. Patients from West Africa or those who have recently been to an area where LF is known to be endemic who have a fever over 38°C (100.4°F) and who are not improving after receiving antimalarial or antibiotic treatment should be believed to have the disease.^5^,^6^.

The first identified case of LF occurred in the Yedseram river valley of Lassa, a remote town in North-Eastern Nigeria and henceforth, the viral disease has been named ‘Lassa fever’^7^. A massive peak in LASV infection occurred in 2016 in Nigeria between January and April with 147 deaths and 268 cases including suspected and confirmed. Infection was found to be spread among 23 of the 36 states in Nigeria. In 2018, an unprecedented upsurge of LF cases occurred in the same region which was later confirmed to be multifactorial and not due to any novel strain. Currently, in the 42nd week of 2022, the predominant age group of the patients affected in Nigeria is 21–30 years. There has been a significant increase in the number of suspected and confirmed cases as compared with 2021 for the same period of the year^8^.

Scanty and sporadic data are available for Nigerian LF for years before 2000 because of a lack of systematic testing. There has always existed a wide gap between the widespread occurrence of the infection in Nigeria and the lack of comprehensive data on the trends of the infection. However, the existing reports suggest that the outbreaks of this fatal disease have ameliorated dimensionally and temporally and are expected to take an even greater catastrophic form for the natives of Nigeria and other parts of West Africa.

The factors contributing to the re-emergence of LF epidemics in Nigeria are nosocomial transmission, travel and migration, the public health system, social factors, effects of civil War and conflicts and the COVID-19 pandemic^9^–^14^. The most promising drug for the treatment of LF is ribavirin. Ribavirin is an analog of guanosine and has a virus-static activity over an array of viruses^15^. The preventive measures that should be taken are – avoiding rodents when people travel to the LF endemic areas, and minimal transmission rates by maintaining strict personal hygiene, isolation of ailing patients, healthcare workers should be careful while transporting body fluids, rigorous washing of hands and other target areas, and use contact and droplet precautions^16^. A combination of convalescent plasma and oral or intravenous ribavirin was administered when the levels of viremia were considerably higher and the levels of aspartate transaminase were higher than 150 IU. Favipiravir is a nucleoside analog that acts as an inhibitor of RNA-dependent RNA polymerase in influenza viruses. It also showed a decrease in viremia. Amodiaquine, niclosamide, zoniporide, arbidol, and apilimod are the drugs that show potential efficacy in the inhibition of the LASV. Arbidol is an anti-influenza drug that inhibits viral fusion with target cells through GP-mediated cell fusion and virus-cell fusion. 5-ethynyl-1-b-D-ribofuranosylimidazole-4-carboxamide (EICAR) and mycophenolic acid target the depletion of viral guanosine-5′-triphosphate and are effective against inosine monophosphate dehydrogenase (IMPDH) and inhibits the replication of LASV at a much lower concentration than ribavirin.

Two such identified compounds are lacidipine and phenothrin which inhibits the entry of the virus by blocking the low-pH-induced membrane fusion. They also inhibited the entry of viruses, the SSP-GP2 interface being the target of entry. The entry if blocked, blocks the replication of the virus and its spread at an early stage^17^. Remdesvir possesses satisfactory inhibitory action against the nucleoprotein of the LASV^18^. Casticinis is a botanical drug that can be used for the therapeutic intervention of LF. Bergamottin, another botanical drug inhibited LASV entry by blocking endocytic trafficking^15^. At present, there are 21 vaccines for LF in the preclinical stages which are developed utilizing cutting-edge technologies rather than a conventional approach, but none of them are approved for LF. The vaccines are inactive or killed viruses and virus-like particles like recombinant stomatitis virus expressing glycoprotein (VSV-LASV-GPC) vaccine, and LASSARAB which is a rabies virus recombinant vaccine, a DNA vaccine, adenovirus vectored vaccines, recombinant vesicular measles virus, vaccinia virus, and ML29 MOPV/LASV live reassorting^19^–^23^ Vaccines that express full-length LASV glycoprotein, provide protection against LF in primates, independent of the nucleoprotein expression, but vaccines expressing only the nucleoprotein or a single glycoprotein gene do not provide any protection. There is no relationship between preexisting high levels of high-titer antibodies and Lassa nucleoprotein and protecting from LF, as there is a primary role of cytotoxic T lymphocytes in protection from LF^24^. LF is seen as a disease of poverty and has a significant impact on those with minimal resources; hence, it is essential that availability of sufficient funding for development of vaccine is crucial. We may currently think of the virus as neglected and endemic to West Africa, but from the insights taken from COVID-19 pandemic are significantly considered then its potential future spread is uncertain to individuals. Therefore, globalization and expanding worldwide travel have been thought to be a possible means of weaponizing the illness. Furthermore, there is a significant amount of uncertainty regarding the role of the environment in the spread of this virus in the West African subregion. Hence, a public awareness campaign in community, state, regional, zonal, and national should be launched to educate the public on the current and potential risk factors associated with the LASV. The correspondence suggests ‘One Health’ approach operational research system to understand the risk factors patterns spatio-geographically, phylogenetic in guiding evidence-based and reservoir(s) mapping, appropriate and adequate time management for efficacious strategic interventions application against the disease demographic threats in Nigeria and the sub-Saharan Africa terrain. To eradicate the illness globally and to prevent re-emerging of LF, technical assistance and financial support, funding across countries, should need to be implicated internationally.

## Ethical approval

Not applicable.

## Sources of funding

None.

## Authors’ contribution

S.M., A.D., J.B., S.S., and S.H.: designed the study. S.M., J.B., S.K., and S.N.: made the first draft. P.S., S.P., and N.M. updated the manuscript. S.M. and S.S.: reviewed the final draft. All authors have critically reviewed and approved the final draft and are responsible for the content and similarity index of the manuscript.

## Conflicts of interest disclosure

The authors declare that they have no financial conflict of interest with regard to the content of this report.

## Research registration unique identifying number (UIN)

Not applicable.

## Guarantor

Sumira Malik and Sarya Swed.
